# Consequences of Daily Administered Parathyroid Hormone on Myeloma Growth, Bone Disease, and Molecular Profiling of Whole Myelomatous Bone

**DOI:** 10.1371/journal.pone.0015233

**Published:** 2010-12-20

**Authors:** Angela Pennisi, Wen Ling, Xin Li, Sharmin Khan, Yuping Wang, Bart Barlogie, John D. Shaughnessy, Shmuel Yaccoby

**Affiliations:** Myeloma Institute for Research and Therapy, University of Arkansas for Medical Sciences, Little Rock, Arkansas, United States of America; Pennsylvania State University, United States of America

## Abstract

**Background:**

Induction of osteolytic bone lesions in multiple myeloma is caused by an uncoupling of osteoclastic bone resorption and osteoblastic bone formation. Current management of myeloma bone disease is limited to the use of antiresorptive agents such as bisphosphonates.

**Methodology/Principal Findings:**

We tested the effects of daily administered parathyroid hormone (PTH) on bone disease and myeloma growth, and we investigated molecular mechanisms by analyzing gene expression profiles of unique myeloma cell lines and primary myeloma cells engrafted in SCID-rab and SCID-hu mouse models. PTH resulted in increased bone mineral density of myelomatous bones and reduced tumor burden, which reflected the dependence of primary myeloma cells on the bone marrow microenvironment. Treatment with PTH also increased bone mineral density of uninvolved murine bones in myelomatous hosts and bone mineral density of implanted human bones in nonmyelomatous hosts. In myelomatous bone, PTH markedly increased the number of osteoblasts and bone-formation parameters, and the number of osteoclasts was unaffected or moderately reduced. Pretreatment with PTH before injecting myeloma cells increased bone mineral density of the implanted bone and delayed tumor progression. Human global gene expression profiling of myelomatous bones from SCID-hu mice treated with PTH or saline revealed activation of multiple distinct pathways involved in bone formation and coupling; involvement of Wnt signaling was prominent. Treatment with PTH also downregulated markers typically expressed by osteoclasts and myeloma cells, and altered expression of genes that control oxidative stress and inflammation. PTH receptors were not expressed by myeloma cells, and PTH had no effect on myeloma cell growth *in vitro*.

**Conclusions/Significance:**

We conclude that PTH-induced bone formation in myelomatous bones is mediated by activation of multiple signaling pathways involved in osteoblastogenesis and attenuated bone resorption and myeloma growth; mechanisms involve increased osteoblast production of anti-myeloma factors and minimized myeloma induction of inflammatory conditions.

## Introduction

Multiple myeloma (MM), a hematologic malignancy of terminally differentiated plasma cells, is closely associated with induction of osteolytic bone disease and skeletal complications in >80% of patients. Myelomatous osteolysis is localized to areas adjacent to tumor growth and is often characterized by increased activity of osteoclasts and suppression of osteoblastogenesis [1]–[3]. Current standard management of MM bone disease is limited to the use of bisphosphonates, which deactivate osteoclasts and may induce adverse side effects such as osteonecrosis of the jaw [4] and impaired renal function [5]. Although bisphosphonates reduce skeletal complications, bone disease often progresses [6], [7], indicating that osteoclastogenesis is only partially inhibited and that suppression of osteoblastogenesis plays a vital role in uncoupling the bone remodeling process in MM [8]–[11].

Recent clinical observations and experimental studies indicate that bone cells are directly involved in survival and expansion of myeloma cells in the hematopoietic bone marrow. While osteoclasts have been shown to promote myeloma cell survival and to protect the cells from spontaneous and drug-induced apoptosis [12]–[14], osteoblasts suppress myeloma cell growth and interfere with osteoclasts' stimulatory effects on myeloma cells [15]. In our mouse model, infusion of mesenchymal stem cells into myelomatous bones was associated with reduced tumor burden [15]. These studies suggest that treating MM with osteoblast-activating agents could simultaneously help control bone disease and myeloma cell growth. Indeed, blocking the Wnt-signaling inhibitor dickkopf-1 (DKK1) with a neutralizing antibody [16], or stimulating Wnt signaling in myelomatous bones by using lithium chloride [17] or Wnt3a [18] resulted in stimulating bone formation and reducing bone loss and myeloma cell growth *in vivo*.

PTH and its biologically active amino-terminal fragments, when given intermittently, can prevent and reverse bone loss in osteoporotic animals and humans [19]–[23]. Recent studies indicate that PTH promotes bone formation primarily by modulating Wnt signaling in bone cells [24]–[32]. Because MM mainly affects elderly people and MM bone disease seems to be a reflection of osteoblast deactivation resulting from myeloma cell secretion of Wnt inhibitors such as DKK1 [16], [33], we hypothesize that daily administered PTH will help control disease progression indirectly by stimulating bone formation.

For our studies, we exploited our SCID-rab and SCID-hu mouse models for primary MM [15], [34]–[37]. These systems are constructed by implanting each SCID mouse with a nonfetal rabbit bone (SCID-rab) or a fetal human bone (SCID-hu) into which primary human myeloma cells are directly injected. The two models are identical in terms of supporting tumor growth and of myeloma-induced bone disease: in both systems, myeloma cells from approximately 80% of patients are successfully engrafted and grow restrictively in the implanted bones, and their growth is characterized by increased levels of human monoclonal immunoglobulins (hIg) in mice sera (indicative of tumor growth) and by induction of severe osteolytic bone disease [16], [34]. To examine the effects of PTH on a large number of patient samples, we used the SCID-rab model because it is more cost-effective and it conveniently allows construction of a large number of animals. We used the SCID-hu system and human global gene expression profiling (GEP) to shed light on molecular mechanisms associated with the effects of PTH on MM bone disease and tumor growth.

## Results

### Hg Myeloma Cell Line Growth is Attenuated and Bone Formation is Stimulated in SCID-rab and SCID-hu Mice after PTH Treatment

Using a procedure we previously reported [38], we established a novel myeloma cell line, Hg, capable of sequential passaging in our animal models. Similar to primary myeloma cells, the Igλ Hg myeloma cells are incapable of growth when cultured alone or cocultured with supporting stromal cells; they also have GEP signatures similar to those of the original patient's plasma cells, are molecularly classified [39] in the MMSET subtype, and express DKK1 (important in MM-induced bone disease) [33]. These observations emphasize the authenticity and clinical relevance of the Hg myeloma line.

**Figure 1:**
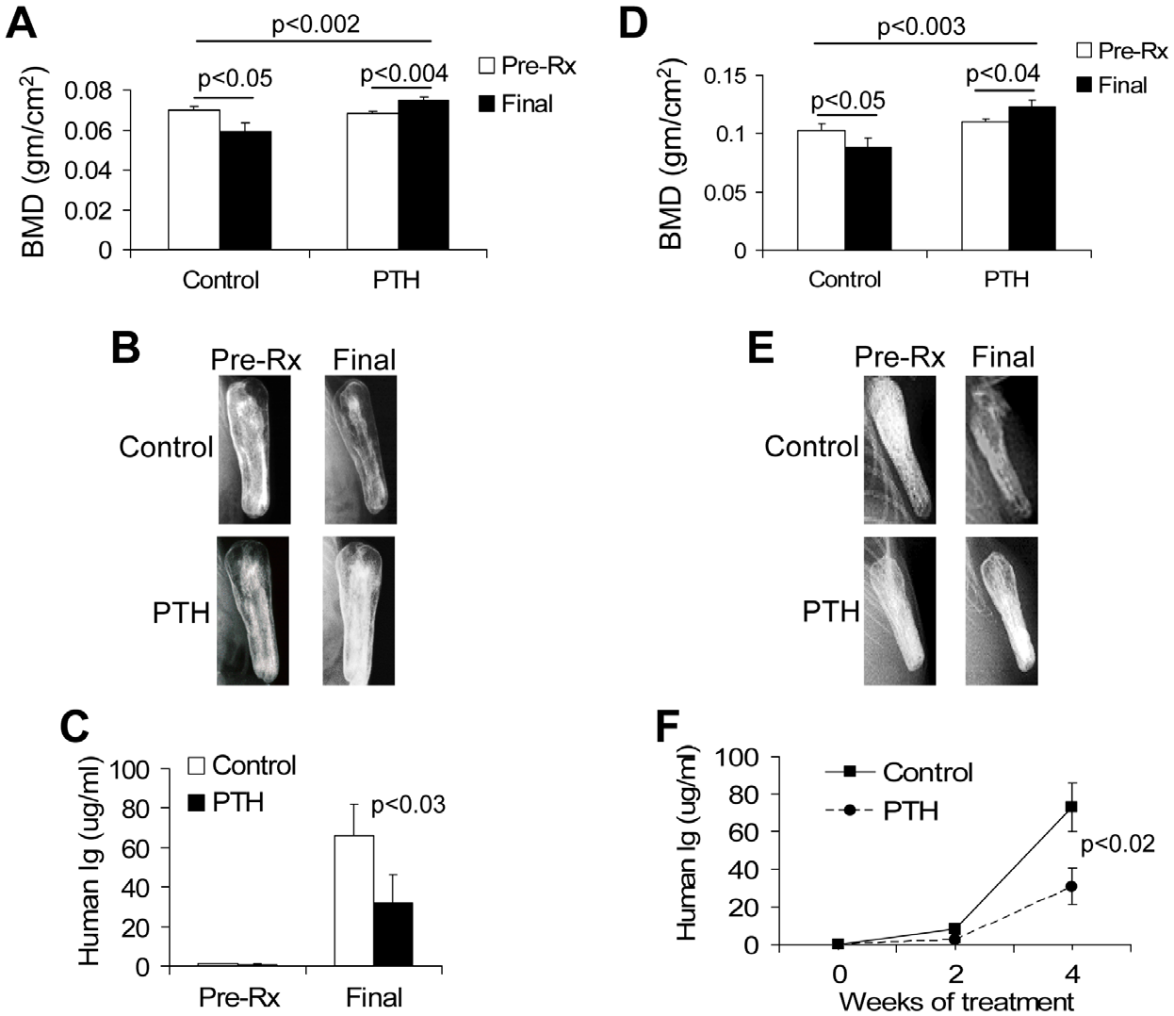
PTH treatment promotes bone formation and attenuates Hg myeloma cell growth in SCID-rab and SCID-hu mice. SCID-rab and SCID-hu mice were engrafted with the Hg myeloma cell line. Upon establishment of MM growth, SCID-rab mice (10 hosts/group) and SCID-hu mice (eight hosts/group) were subcutaneously treated with saline or PTH (80 µg/kg/d) for 4 weeks. (A–C) Effects of PTH in the SCID-rab model: changes in bone mineral density (BMD) levels of the implanted bone (A), representative X-ray radiographs before initiation of treatment (Pre-Rx) and at experiment's end (Final) (B), and MM burden determined by measuring levels of circulating human immunoglobulins (hIg) before initiation of treatment (Pre-Rx) and at experiment's end (Final) (C). (D–F) Effects of PTH in the SCID-hu model: changes in BMD levels of the implanted bone (D), representative X-ray radiographs before initiation of treatment (Pre-Rx) and at experiment's end (Final) (F), and levels of hIg before initiation of treatment (Pre-Rx) and at experiment's end (Final) (E).

We used Hg cells, along with the SCID-rab and SCID-hu model systems for MM [34], [35], to characterize the effects of PTH on bone metabolism and on myeloma growth in myelomatous bone and to shed light on the molecular mechanisms of PTH. SCID-rab (10 mice/group) and SCID-hu (eight mice/group) mice engrafted with Hg myeloma cells were treated with saline (as a control) or PTH for 4 weeks. Whereas bone mineral density (BMD) of the myelomatous bones in saline-treated hosts was reduced by 14±5%, it was increased by 10±2% in PTH-treated hosts (p<0.002 saline vs. PTH-treated hosts); for both treatment groups, effects were similar in SCID-rab and SCID-hu mice. The final BMD values of implanted bones from saline- and PTH-treated SCID-rab mice were 0.059±0.004 and 0.0075±0.016 g/cm^2^, respectively (p<0.002) (**Figure 1A**); in SCID-hu mice, they were 0.088±0.007 and 0.0123±0.05 g/cm^2^, respectively (p<0.003) (**Figure 1D**). X-ray radiographs also demonstrated the bone-anabolic effects of PTH on myelomatous bones from SCID-rab mice (**Figure 1B**) and SCID-hu mice (**Figure 1E**). Positive effects of PTH on preventing MM bone disease and promoting bone anabolism were associated with reduced growth of Hg myeloma cells, which was assessed by measuring hIg in mice sera, in SCID-rab mice (**Figure 1C**) and SCID-hu mice (**Figure 1F**).

**Figure 2:**
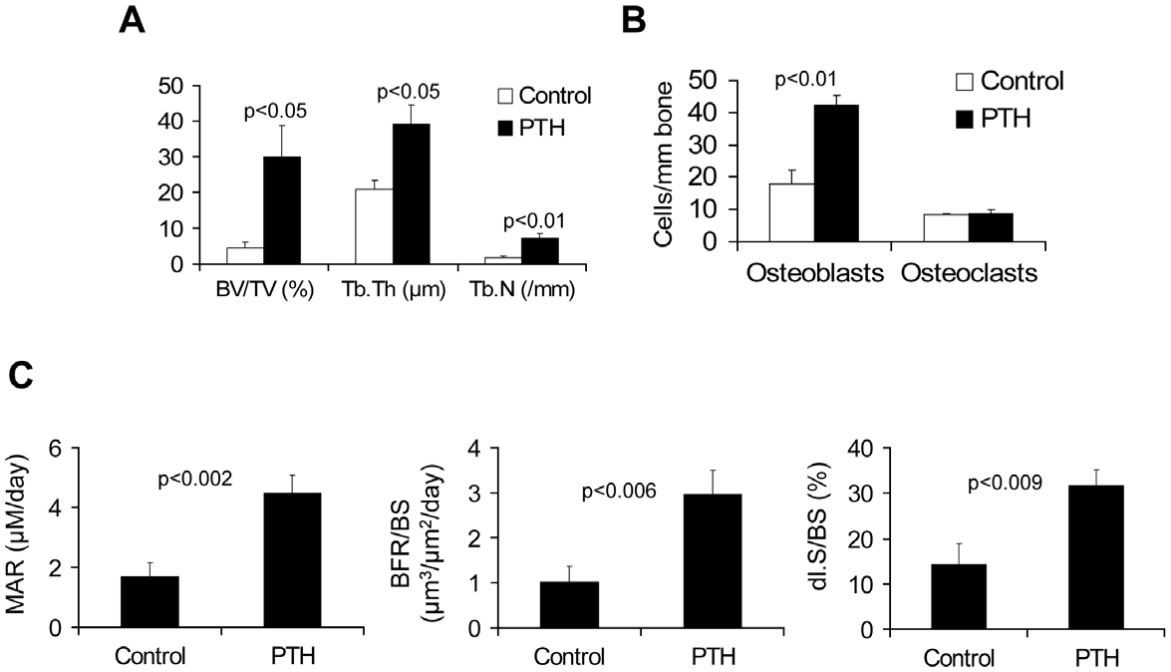
Treatment with PTH promotes bone formation in SCID-rab mice engrafted with Hg myeloma cells. SCID-rab mice were engrafted with Hg myeloma cells. Upon establishment of MM growth, SCID-rab mice (10 hosts/group) were subcutaneously treated with saline or PTH (80 µg/kg/d) for 4 weeks. (A) Static histomorphometry parameters (trabecular bone volume [BV/TV], thickness [Tb.Th], and number [Tb.N]) in myelomatous bones from hosts treated with saline (Control) or PTH. (B) Number of osteocalcin-expressing osteoblasts and tartrate-resistant acid phosphatase (TRAP) -expressing osteoclasts in myelomatous bones from hosts treated with saline (Control) or PTH. (C) Dynamic histomorphometry parameters (mineral apposition rate [MAR], bone formation rate/bone surface [BFR/BS], and double-labeled surface/bone surface [dl.S/BS]) in myelomatous bones from hosts treated with saline (Control) or PTH.

After PTH treatment, static histomorphometric analyses of myelomatous implanted bones from SCID-rab mice revealed increased BV/TV (p<0.05), Tb.Th (p<0.05), and Tb.N (p<0.01) (**Figure 2A**). Implanted bone sections from SCID-rab hosts treated with saline or PTH were immunohistochemically stained for osteocalcin and histochemically stained for tartrate-resistant acid phosphatase (TRAP). Analysis revealed that PTH treatment resulted in a marked increase in the number of osteocalcin-expressing osteoblasts (p<0.01) and no change in the number of TRAP-expressing osteoclasts (**Figure 2B**). Furthermore, dynamic histomorphometry in these bones revealed that PTH treatment resulted in marked increases in mineral apposition rate (MAR, p<0.002), double-labeled surface (dl.s/BS, p<0.009), and bone-formation rates (BFR, p<0.006) (**Figure 2C**). The findings indicate that PTH promotes bone anabolism in myelomatous bones.

### Primary Myeloma Growth is Attenuated and Bone Formation is Stimulated in SCID-rab Mice after PTH Treatment

SCID-rab mice were successfully engrafted with primary myeloma cells from 10 patients. Myeloma cells were taken from patients who varied in clinical disease stage and bone disease status (**Table 1**); most were newly diagnosed and their samples were selected for the study solely based on availability of tumor cells. For this set of experiments, cells from the same patient were injected into two mice, and the host with higher hIg levels (indicative of higher tumor burden) received PTH treatment, while the other received saline treatment. Upon establishment of MM tumor growth (indicated by hIg level >10 µg/ml), 10 SCID-rab hosts engrafted with myeloma cells from 10 patients were treated with PTH for 4 weeks; an additional 10 matching SCID-rab hosts served as controls and were treated with saline for 4 weeks. As previously shown [34], [35], different patients' myeloma cells produced different MM growth patterns.

**Table 1:** Patient characteristics and changes in BMD of the implanted bone and hIg levels in SCID-rab mice during the experiment.

Pt.	Stage*	Prior Treatment	Isotype	MRI FL**	Bone Disease¶	BMD (% of pre-Rx)±		hIg (µg/ml)#	
						Cont	PTH	Cont	PTH
1	IIIa	NO	IgG λ	YES	YES	86	110	219	90
2	IIIa	NO	IgA λ	YES	ND$	96	104	1173	289
3	III	NO	IgG κ	YES	YES	106	175	183	12
4	IIIa	YES	IgG κ	YES	YES	89	110	252	92
5	III	NO	IgG λ	YES	NO	54	88	550	379
6	IIIa	NO	IgG κ	YES	YES	79	110	387	333
7	IIIa	NO	IgG κ	YES	YES	83	101	335	69
8	IIIa	NO	IgG κ	YES	NO	67	128	489	475
9	IIIa	NO	IgG λ	YES	YES	75	111	530	440
10	IIIa	NO	IgG κ	YES	YES	30	84	440	450

*Stage at diagnosis, according to the Durie-Salmon staging system.

**Existence of focal lesions (FL) determined by magnetic resonance imaging (MRI).

¶ Existence of lytic bone lesions determined by standard X-rays.

± BMD of the implanted bone determined by DEXA and calculated as percent of pretreatment level.

# Circulating hIg in mice sera determined by ELISA and calculated as percent of pretreatment level.

$ Not done.

**Figure 3:**
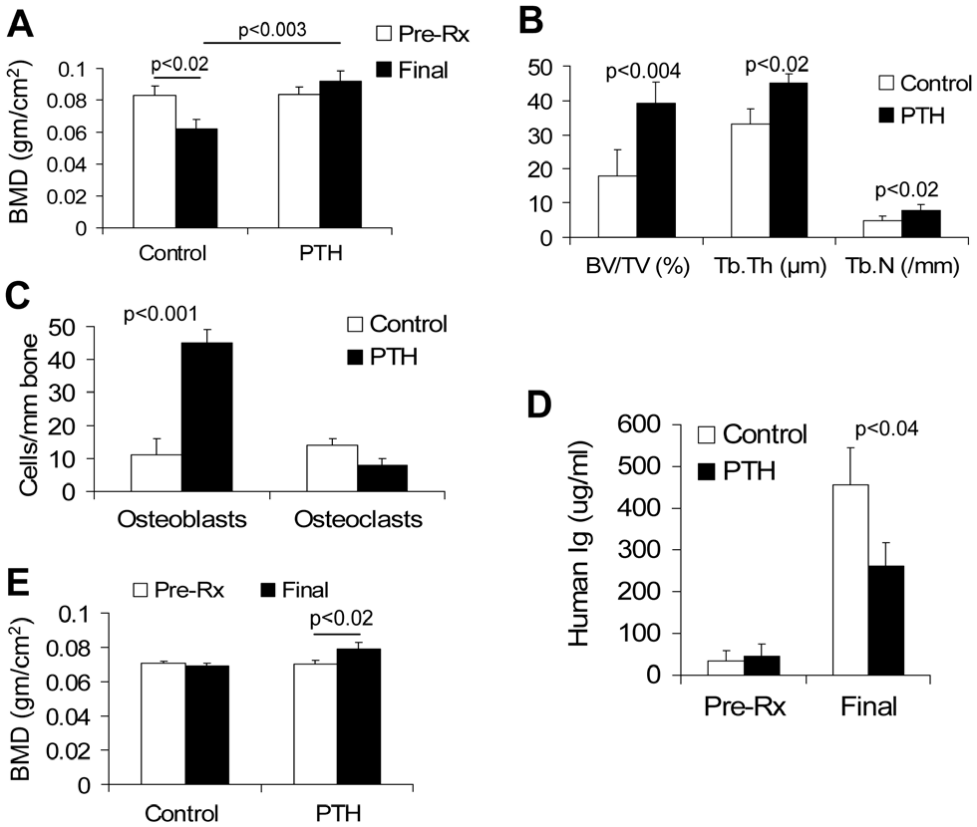
PTH treatment promotes bone formation and attenuates growth of primary myeloma cells in SCID-rab mice. SCID-rab mice were engrafted with primary myeloma cells from 10 patients. In this set of experiments, myeloma cells from the same patient were injected into two mice; one host was treated with saline (Control) and the other with PTH for 4 weeks. A total of 10 hosts were used in each group (see also Table 1). (A) Level of BMD of the implanted bone before initiation of treatment (Pre-Rx) and at experiment's end (Final). (B) Static histomorphometry parameters (BV/TV, Tb.Th, Tb.N) in myelomatous bones from hosts treated with saline (Control) or PTH. (C) Number of osteocalcin-expressing osteoblasts and TRAP-expressing osteoclasts in myelomatous bones from hosts treated with saline (Control) or PTH. (D) Human immunoglobulin (hIg) levels (surrogate for myeloma tumor burden) before initiation of treatment (Pre-Rx) and at experiment's end (Final) in SCID-rab mice engrafted with Hg myeloma cells. (E) Level of BMD of the uninvolved murine femur before initiation of treatment (Pre-Rx) and at experiment's end (Final).

Implanted myelomatous bones had higher BMD after treatment with PTH than before treatment in six experiments, and bone loss was less severe in one experiment (**Table 1**). Overall, analysis of pooled results of experiments with all 10 patients' myeloma cells demonstrated that BMD of the myelomatous bone was 24±7% lower than pretreatment levels in saline-treated hosts (0.062±0.006 g/cm^2^ versus 0.083±0.006 g/cm^2^ pretreatment level, p<0.02), but it was 12±8% higher than pretreatment levels in the PTH-treated group (0.092±0.006 g/cm^2^ versus 0.084±0.005 g/cm^2^ pretreatment level); the difference in final BMD values of implanted bones from saline- and PTH-treated hosts was statistically significant (p<0.003) (**Figure 3A**). Static histomorphometric analyses comparing myelomatous implanted bones before and after treatment with either saline or PTH demonstrated increased BV/TV (p<0.004), Tb.Th (p<0.02), and Tb.N (p<0.02) after treatment with PTH (**Figure 3B**). Treatment with PTH increased the number of osteocalcin-expressing osteoblasts (p<0.001) and had no effect on the number of TRAP-expressing osteoclasts (**Figure 3C**). Although PTH treatment had heterogeneous effects on MM growth (**Table 1**), the overall tumor burden (final hIg levels) was significantly lower (p<0.04) in PTH-treated hosts than in saline-treated hosts (**Figure 3D**). These data indicate that PTH effectively prevents MM-associated bone disease, promotes bone formation, and reduces MM growth in hosts engrafted with primary myeloma cells from different patients, which corresponds to the results seen in similar experiments with the Hg myeloma cell line.

**Figure 4:**
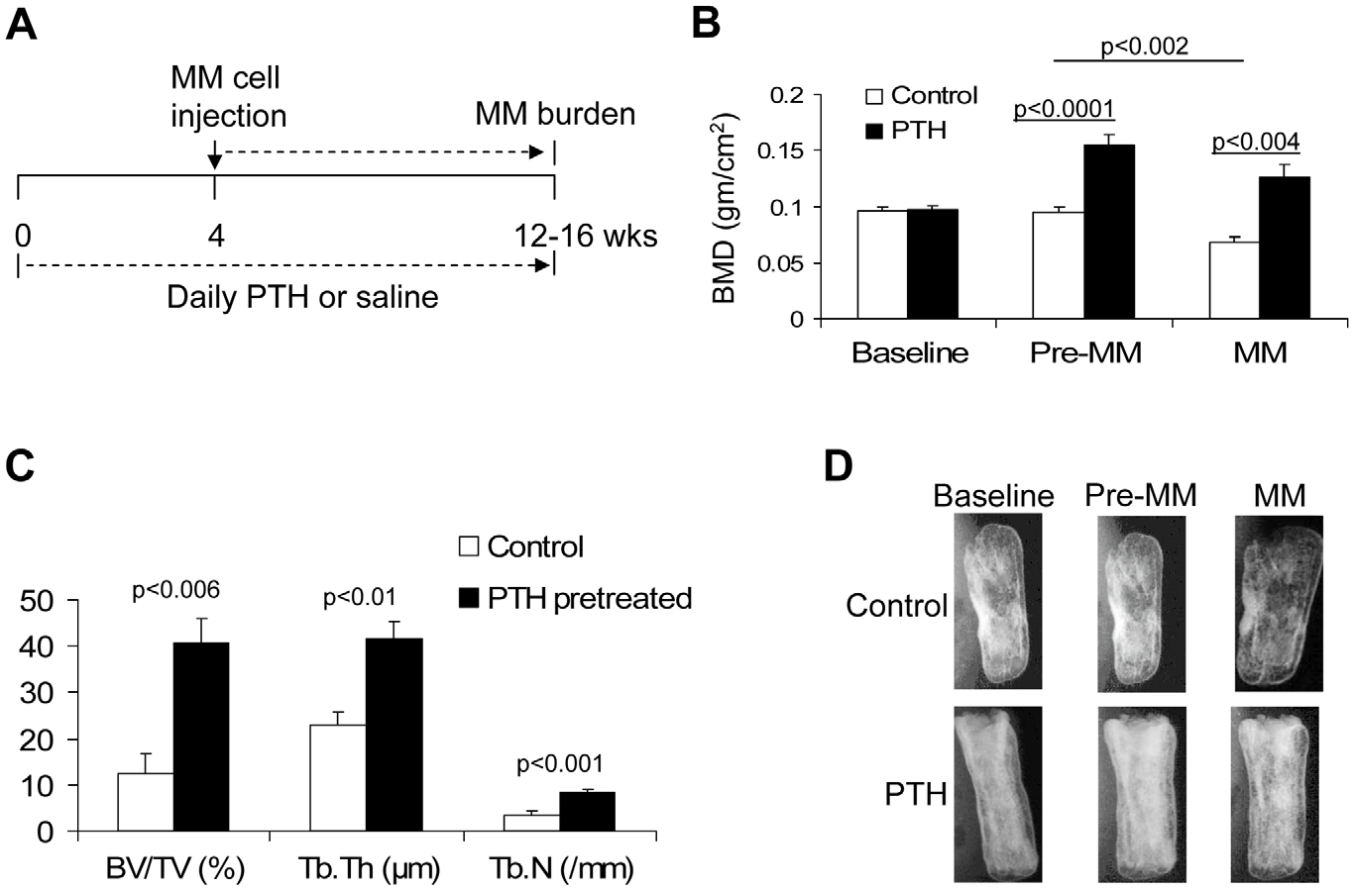
Bone formation induced by PTH pretreatment inhibits myeloma bone disease. SCID-rab mice (15/group) were treated with PTH or saline for 4 weeks and then were injected with myeloma cells (BN stroma-dependent myeloma cells, six mice/group; or myeloma cells from one of three patients, three mice/group for each patient's cells). (A) A schema demonstrating the experimental design. (B) BMD levels of the implanted bones were measured before PTH treatment was initiated (Baseline); after 4 weeks of PTH treatment, just before myeloma cell injection (Pre-MM); and 8–12 weeks after myeloma cell injection (MM). Note that PTH treatment increased BMD levels of implanted bones before inoculation with myeloma cells (Pre-MM), an effect that was retained after engraftment of MM in these bones (MM). (C) Static histomorphometric analysis of the implanted bones at the end of the experiment. (D) Representative X-ray radiographs of implanted bones at different stages of the experiments in three hosts treated with saline (Control) or PTH. Note that bone mass increased after treatment with PTH (Pre-MM) and that bone loss after MM engraftment was less profound in the PTH-pretreated group.

Treatment with PTH also resulted in marked BMD increases (>12%; p<0.02) from pretreatment levels in uninvolved murine femurs of SCID-rab hosts (**Figure 3E**) and BMD increases in nonmyelomatous implanted rabbit bones of SCID-rab mice (see below **Figure 4B**, “Pre-MM,” p<0.0001).

### Pretreatment with PTH Prevents MM Progression

The effects of PTH pretreatment on tumor progression were examined in SCID-rab mice. Mice were treated with PTH for 4 weeks before and after inoculation with BN stroma-dependent myeloma cells [38] (engineered to express luciferase; six mice/group) or with primary myeloma cells from three patients (for each patient's cells, a total of six hosts were used: three pretreated with saline, three pretreated with PTH). The effects of PTH pretreatment on tumor growth were monitored for 8–12 weeks after inoculation with myeloma cells (see schema, **Figure 4A**). Treatment with PTH did not stop after inoculation of the myeloma cells, in order to prevent a potential increase in osteoclast activity and bone resorption after PTH withdrawal. The main goal of this study was to test the ability of PTH pretreatment to prevent bone loss during MM progression. After 4 weeks of PTH treatment (before injection of myeloma cells), BMD of the implanted nonmyelomatous rabbit bones was 60±16% higher than levels before PTH treatment (p<0.0001) (**Figure 4B**). After myeloma cell engraftment, BMD of the bones implanted in saline-treated hosts, but not in PTH-treated hosts, was significantly lower than (29±8%) before myeloma cells were injected (p<0.002). At the end of the experiments, BMD of the implanted bone in PTH-treated hosts was slightly lower than before myeloma cells were injected, but it was significantly higher than in saline-treated hosts (p>0.004, **Figure 4B**). In the PTH-pretreatment group, the implanted bones had significantly higher BV/TV (p<0.006), Tb.Th (p<0.01), and Tb.N (p<0.001) than implanted bones from the control group, based on static histomorphometry (**Figure 4C**). The consequences of PTH pretreatment on bone mass before and after myeloma cell inoculation were also visualized on X-ray radiographs (**Figure 4D**).

**Figure 5:**
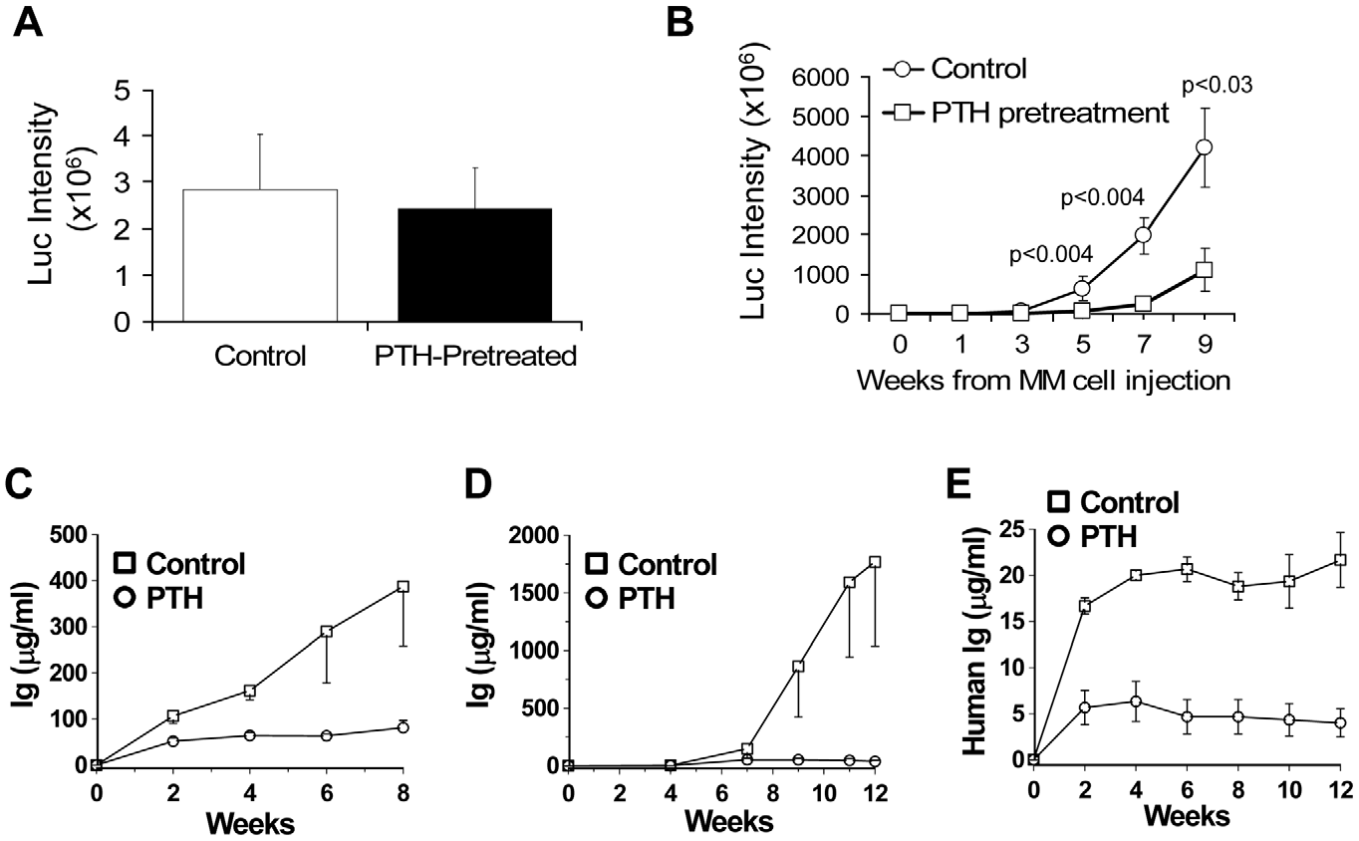
PTH pretreatment inhibits myeloma progression. SCID-rab mice were pretreated with PTH for 4 weeks and then injected with BN stroma-dependent myeloma cells (six mice/group) or with myeloma cells from one of three patients (for each patient's cells, a total of six hosts were used: three pretreated with saline, three pretreated with PTH). Treatment with PTH continued throughout the experimental period (see schema of the experimental design in Figure 4A). (A) *In vivo* live-animal imaging revealed similar luciferase (Luc) activity in BN myeloma cells a few hours after injection into saline- and PTH-pretreated hosts, indicating that similar numbers of myeloma cells were injected into the implanted bones in both groups. (B) Pretreatment with PTH inhibited growth of BN myeloma cells in SCID-rab mice. (C–E) Pretreatment with PTH delayed growth of primary myeloma cells from three different patients.

Because PTH pretreatment resulted in increased bone mass, it is possible that myeloma cell injection into these bones was less efficient and resulted in fewer cells reaching the bones, which would contribute to the appearance of an antimyeloma effect. To investigate this possibility, luciferase-expressing myeloma cells were used to compare the number of myeloma cells that were injected into implanted bones with and without PTH pretreatment. We previously demonstrated that luciferase intensity highly correlated with myeloma cell numbers *in vitro* and *in vivo* [38]. The luciferase assay demonstrated that the numbers of myeloma cells injected into the implanted bones of PTH-pretreated hosts were similar to those injected into saline-pretreated hosts (**Figure 5A**).

Pretreatment with PTH significantly inhibited the growth of luciferase-expressing BN myeloma cells in SCID-rab mice (six mice/group) 5 weeks (p<0.004), 7 weeks (p<0.004), and 9 weeks (p<0.03) after inoculation with myeloma cells (**Figure 5B**). Although BN cells differ from Hg cells in their ability to grow in coculture with stromal cells, *in vitro* growth of BN cells was reduced to a greater extent in coculture with osteoblasts than with stromal cells (data not shown), suggesting that the growth of BN cells *in vivo*, like that of Hg cells, is affected by increased osteoblast activity. Furthermore, pretreatment with PTH resulted in delayed growth of myeloma cells taken from three additional patients (**Figure 5C–E**). Taken together, our data indicate that PTH pretreatment effectively increased bone mass, and this effect was associated with inhibition of myeloma cell engraftment and MM progression.

### Global GEP Revealed Molecular Mechanisms Associated with PTH Effects in Myelomatous Bone

**Figure 6:**
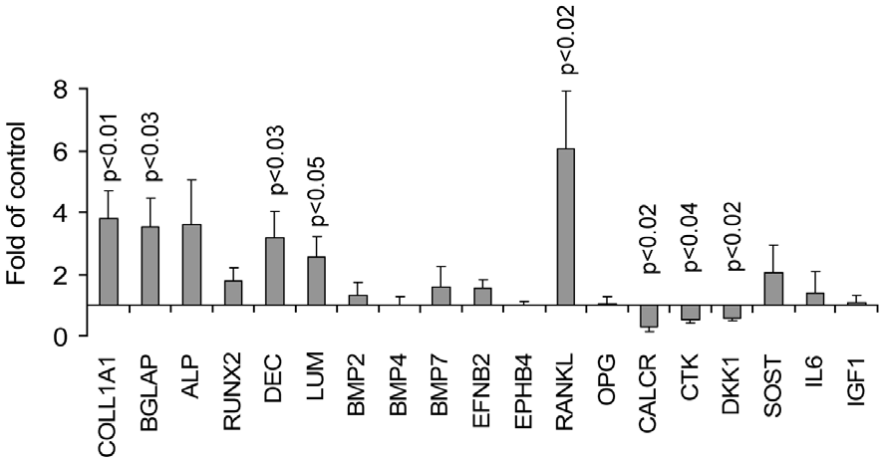
PTH treatment alters gene expression in whole myelomatous human bones engrafted with Hg myeloma cells. SCID-hu mice engrafted with Hg myeloma cells were treated with saline or PTH for 4 weeks. Mice were sacrificed 2 hours after the last injection, and RNA was extracted from the whole myelomatous human bone (five bones/group). RNA samples were subjected to global gene expression profiling (see partial list in Table 2 and complete list in Table S1 of differentially expressed genes). The same RNA samples were also used to validate and analyze expression of selected bone-associated genes by qRT-PCR as indicated in the figure.

We exploited the SCID-hu system to shed light on molecular mechanisms involved in the effects of PTH treatment on bone remodeling and myeloma growth. Total RNA was extracted from human bones of SCID-hu mice engrafted with Hg myeloma cells and treated with saline or PTH for 4 weeks. These RNA samples were subjected to global GEP using the human Affymetrix U133-Plus microarray [39]. Treatment with PTH resulted in significantly altered (≥2-fold) expression of 753 genes in myelomatous bones; 343 genes were upregulated and 410 genes were downregulated (**Table S1**). Expression of 19 genes was also evaluated by quantitative real-time polymerase chain reaction (qRT-PCR); the results supported those of GEP (**Table 2** **,** **Figure 6**).

**Table 2:** Selected genes whose expression was upregulated or downregulated in whole myelomatous human bone by PTH treatment*.

Functional Category	Upregulated Genes	Downregulated Genes
**Osteoblast associated**	*BGLAP, PTN, THBS4, COL11A1, SMOC2, SPARC, THBS2, LUM, COL5A2, COL14A1, RUNX2, SGCD, GJA1, COL12A1, COL5A1, CDH11, VCAN, BGN, COL8A2, NID2, COL21A1, COL3A1, THBS1, POSTN, COL6A3, DCN, GPC1, COL6A1*	
**Osteoclast associated**	*TNFSF11*	*CA5BL, ATP6V0E1, NFATC1, ACP5*
**Myeloma plasma cell associated**		*CD38, ITGB7, AURKB, STAT3, WHSC1, IRF4, FGFR3, IGL@*
**cAMP/PKA**	*NR4A3, RGS2, RGS1, PDE7B, PDE3A, PDE4D, FOS, CREB3L1, FOSL2, JUNB, PDE3B*	
**Wnt signaling**	*WNT5A, ROR2, SPON1, CYR61, TWIST1, LRP4, FZD1, WISP1, NKD2, TCF4, PPARD*	*DKK1, CTBP1, TNIK*
**FOXO and oxidative stress**	*FOXC1, MSX1, MSX2, FOXF1*	*FOXO3, CAT, FOXO1A*
**Cytokines and chemokines**	*CXCR7, CXCL14, ANGPTL2, IGFBP5, IGFBP7, ANGPTL4, ANGPT2*	*ANGPT1, EGFR, VEGFB, CCL5, IL17RB, PECAM1, CXCR3*
**MMPs**	*MMP13, MMP2*	
**Prostaglandins**	*PTGES, PTGFRN*	
**Integrins**	*ITGBL1, ITGA11, ITGB5, ITGAV*	*ITGB2, ITGA2B*
**Inflammation regulators**	*TNFAIP6*	*AIF1*
**Bone associated signaling**	*FGFR2, FGFR1, ID4, SMAD6, ID3, TGFBR1, TGFB2, PDGFA, PDGFRA, HGF*	
**Phosphatases**	*PTPRD, DUSP10, PTPRS, PTPRF, PPAP2A*	*PTPN6, SIRPA, PPP1R11, PTP4A1, PPP3CC*
**G-protein**	*GPR158, GPRC5C, GPR153*	*GPRC5D*

*SCID-hu mice engrafted with Hg myeloma cells were treated with saline or PTH for 4 weeks. Mice were sacrificed 2 hours after the last injection. RNA extracted from the whole myelomatous human bone (five bones/group) was subjected to global gene expression profiling. Genes are listed in order based on fold changes compared to the saline-treated group. For detailed information, see Table S1.

Many of the genes demonstrated by GEP to have significantly altered expression after PTH treatment are relevant to bone remodeling, PTH signaling, and myeloma pathogenesis (**Table 2**). There was a striking upregulation of osteoblastic bone matrix genes. PTH-induced upregulation of *TNFSF11* (RANKL) and unchanged or reduced expression of important osteoclast-associated genes (e.g., *ACP5*/TRAP, *CTSK*) confirmed our observation that osteoclast numbers were unchanged in histological analyses of myelomatous bone sections (see **Figure 2B** **,** **Figure 3C**). A number of MM-associated genes were downregulated by PTH treatment, including *CD38*, *IGL*, and genes known to be expressed in MMSET-type MM (e.g., *FGFR3*, *WHSC1*, *ITGB7*), which is the disease subtype from which the Hg cell line was derived. These GEP observations support the findings that PTH promotes bone anabolism, does not increase osteoclast activity, and attenuates growth of myeloma cells in bone.

The GEP data also highlighted alterations in signaling associated with PTH treatment in myelomatous bone. Treatment with PTH affected expression of various genes that regulate cAMP signaling, Wnt signaling, and various forkhead box transcription factors, along with altered expression of cytokines (e.g., *ANGPT1*, *ANGPT2*), chemokines (*CXCL14*), growth factors (*TGFbeta2*, *PDGFA*), and receptors (*TGFBR1*, *PDGFRA*). Inflammatory-associated genes such as *TNFAIP6* and *AIF1* were upregulated and downregulated, respectively, after PTH treatment. Noteworthy are results showing no significant alteration in expression of *SOST* but downregulation of *DKK1*, both of which have been implicated in the bone-anabolic mechanism of PTH. Taken together, these findings suggest that PTH treatment affects multiple signaling pathways known to play significant roles in bone remodeling.

### Myeloma Cells do not Express PTH Receptors, and PTH has no Effect on Their Growth *In Vitro*

**Figure 7:**
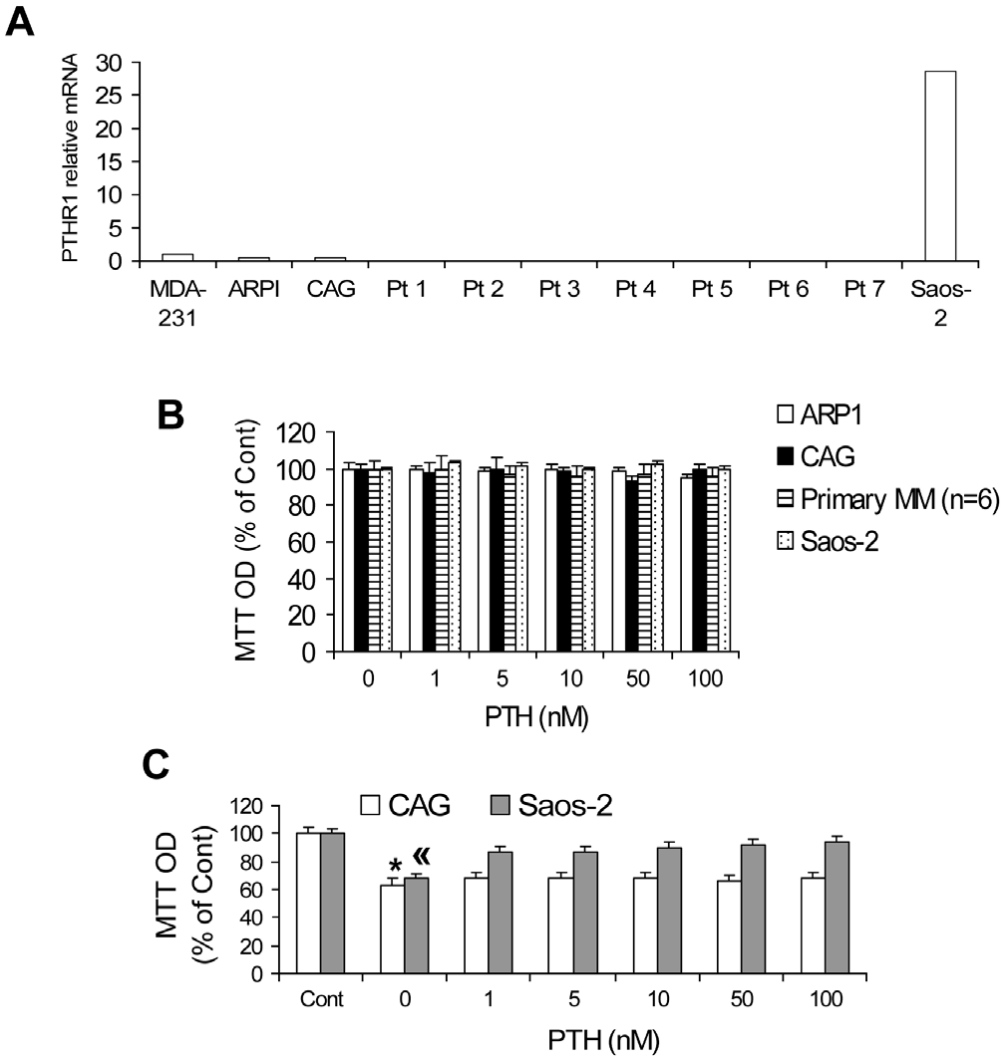
Myeloma cells do not express PTH receptors; PTH does not affect myeloma cell growth *in vitro*. (A) Expression of the type-1 PTH receptor (PTH1R) was determined by qRT-PCR. Saos-2 osteosarcoma cells [74] and MDA-231 breast cancer cells [73] were used as positive and negative controls, respectively, for PTH1R expression. Note that myeloma cell lines and primary myeloma cells from seven patients did not express PTH1R. In addition, type-2 PTH receptor was not detected in any myeloma cell samples, but it was highly expressed in the positive control (human brain tissue; data not shown). (B) Myeloma cell lines (ARP1 and CAG), primary myeloma plasma cells from six patients, and Saos-2 osteosarcoma cells were cultured in serum-containing medium and treated with various concentrations of PTH. Effects on cell growth were determined by MTT (3-(4,5-dimethylthiazol-2-yl)-2,5-diphenyltetrazolium bromide) assay. Primary myeloma cells were cultured for 24 hours with PTH; all cell lines were cultured for 72 hours with PTH. Note that growth of cells was not affected by PTH. (C) CAG and Saos-2 cells were cultured in serum-free medium and treated with various concentrations of PTH for 72 hours before being subjected to MTT assay. Note that Saos-2 cells, but not CAG myeloma cells, were protected from serum starvation-induced growth inhibition. *p>0.001 versus saline-treated cells (Cont); « p<0.0001 versus Cont and all PTH-treated groups.

In two myeloma cells lines (CAG and ARP1) and primary myeloma plasma cells from seven patients, qRT-PCR was used to confirm that type-1 PTH receptor (PTH1R) was not expressed by myeloma cells (**Figure 7A**) [40]. qRT-PCR also demonstrated that expression of type-2 PTH receptor was undetectable in all tested myeloma cell samples, although it was highly expressed in human brain tissue (data not shown). Global GEP data in our institute confirmed the lack of PTH receptor expression in >40 myeloma cell lines, including Hg and BN myeloma cell lines. PTH had no effect on *in vitro* growth of myeloma cell lines or primary myeloma plasma cells (n = 6) in the presence (**Figure 7B**) or absence (**Figure 7C**) of serum. However, control cells (Saos-2 osteosarcoma cells) that express PTH1R [41] were protected from serum starvation-induced growth inhibition when incubated with PTH (**Figure 7B**), and no effect was observed in serum-containing medium (**Figure 7C**).

## Discussion

In this study, we demonstrated that PTH is capable of increasing bone mass in myelomatous bones *in vivo* and that the increased bone formation is associated with a concomitant reduction in growth of the Hg myeloma cell line and primary myeloma cells from certain patients. In our animal model, pretreatment with PTH also resulted in increased bone mass and a significant delay in MM progression. Treatment with PTH markedly increased the number of differentiating osteoblasts, but the number of osteoclasts remained unchanged in bones engrafted with Hg myeloma cells and was moderately reduced in bones engrafted with primary myeloma cells. Strongly supporting our findings, GEP analyses of whole myelomatous bones showed increased expression of osteoblastic markers and reduced expression of osteoclastic and myeloma cell markers. GEP analyses also provided insight on molecular mechanisms that mediate the various effects of PTH in myelomatous bones. Because PTH had no direct effects on growth of myeloma cells, we conclude that shifting bone turnover to an anabolic state in myelomatous bone results in negative effects on MM progression. The results of this study support our previous findings, and those of others, that increased bone mass resulting from exogenous MSC cytotherapy [15] or treatment with DKK1-neutralizing antibody [15], Wnt3a [18], or lithium chloride [17] negatively impact MM tumor burden in bone.

PTH is approved for treatment of osteoporosis in men and women [21], [22], but patients with cancer currently are not treated with PTH because of concerns that the treatment might promote tumor growth or osteosarcoma [42]. In the present study, we tested the effect of a relatively high dose of PTH (80 µg/kg/d) on MM bone disease and tumor growth in our animal models. Similar high doses had previously been tested in animal models for osteoporosis [20], [31], [43]. Although analogy to the clinical setting cannot properly be made due to the significantly higher metabolic rate of mice compared to humans, it is of interest to test whether lower doses of PTH have a significant effect on prevention of MM bone disease. Our study demonstrated not only that PTH has no direct stimulatory effects on myeloma cells but also, intriguingly, that PTH has antitumor properties, presumably due to its ability to alter the bone marrow microenvironment. Although PTH has been shown to promote osteoclastogenesis in certain (but not all) physiological and experimental conditions [20], [23], [43], the numbers of osteoclasts in myelomatous bones in our study did not increase during the experimental period. MM-related osteolysis results from an uncoupling of the processes of osteoclastic bone resorption and osteoblastic bone formation, which causes bone remodeling to shift toward bone destruction as activities of osteoclasts increase and of osteoblasts decrease [1], [2]. We speculate that, in these conditions, PTH contributes to restoring balance to the coupled bone-remodeling process in MM, which results in increasing the number of bone-building osteoblasts without altering the number of osteoclasts and, in some cases, even reducing the number of osteoclasts.

Indeed, GEP analysis demonstrates alterations in multiple signaling pathways that are critically involved in bone remodeling and in regulating the coupling of bone formation and bone resorption. To our knowledge, this is the first GEP analysis on human bones following treatment with PTH, and it may provide insight into additional mechanisms involved with this hormone's effects on bone. Interestingly, many of the differentially expressed genes are not expressed by the Hg myeloma cells and are thus considered microenvironment-associated genes.

The actions of PTH are mediated by a G protein-coupled receptor, PTH receptor 1 (PTHR1) [44]. When PTH binds to the receptor, Gα_s_-mediated activation of adenyl cyclase is stimulated, which then stimulates cAMP production and subsequent activation of protein kinase (PKA). Confirming activation of the cAMP/PKA pathway in myelomatous bones after PTH treatment, GEP analyses showed upregulation of cAMP-specific phosphodiesterases (e.g., *PDE4D*), which are involved in regulating osteoblast production of PTH-induced cAMP [45]. GEP also revealed upregulation of genes known to be stimulated by PTH treatment and/or associated with osteoblast differentiation, including *RUNX2*, *FOS*, *JUNB*, *NR4A2*, *NR4A3*, *RGS2*, *GJA1* (connexin43), *MMP13* (collagenase-3), *BGLAP* (osteocalcin), *SPARC* (osteonectin), *DCN* (decorin), *LUM* (lumican), *BGN* (byglican), and various collagen types [46], [47].

### Wnt Signaling

Although PTH is known to stimulate bone formation, the underlying mechanisms are not yet fully understood [48]. Several studies have explored links between PTH and the Wnt signaling pathway [24]–[32], and our data confirm that Wnt signaling is involved in the anabolic activity of PTH in myelomatous bones. We identified increased expression of genes encoding several components of the canonical Wnt signaling pathway and target genes of the pathway, such as *FZD1*, *TCF4*, *AXIN2*, *NKD2*, *TWIST1*, *WISP1*, and *CYR61*; we also noted downregulation of negative regulators of the Wnt pathway (i.e., *CTBP1*, *KREMEN2*, and *DKK1*). Although reduced levels of two other Wnt signaling inhibitors, sclerostin (*SOST*) and secreted frizzled related protein-2 (*FRZB*), have been described in response to PTH [25], [27], [29], we did not detect changes in expression of those genes in our experimental setting.

The significant downregulation of *DKK1* that resulted from PTH treatment emphasizes the critical role of this factor in myeloma-induced suppression of osteoblastogenesis [16], [33]. Because *DKK1* is expressed by Hg myeloma cells and PTH treatment resulted in reduced growth of these cells in myelomatous bones, *DKK1* downregulation could be a result of direct effects of PTH on osteoblasts, it could be an epiphenomenon of reduced tumor burden, or it could be a combination of both. Our data are consistent with recent studies demonstrating that PTH suppresses osteoblast production of DKK1 and that PTH can stimulate Wnt signaling and bone anabolism in the presence of DKK1 [26], [49]. Interestingly, PTH reduced expression of *LRP4*, which has been suggested to act as a sink and compete with Lrp5/6 for the binding of soluble Wnt antagonists (e.g., Wise, DKK1, and sclerostin) that are then not available to suppress the signal through the Lrp5/6 axis [50]. These findings strongly indicate the important role of canonical Wnt signaling in the bone-anabolic effects of PTH.

Recent evidence shows that WNT5A activation of noncanonical Wnt signaling stimulates the osteogenic properties of human osteoblastic cells by homodimerization and activation of ROR2 [51]. We found that *WNT5A* and *ROR2* are significantly upregulated by PTH treatment, suggesting a role for noncanonical Wnt signaling in the anabolic effects of this hormone.

Recent studies suggest that bone-anabolic effects of PTH are amplified by bone marrow T lymphocytes secreting Wnt ligand Wnt10b [31]. In our experimental system, *Wnt10b* was expressed at very low levels in the human bones, and its expression was not affected by PTH treatment. Our data showed that PTH also increased BMD of the uninvolved femurs of SCID mice, which are deficient of T and B lymphocytes, suggesting that PTH effectively stimulates Wnt signaling in the absence of T lymphocytes.

### Other Signaling Pathways

PTH resulted in upregulation of *FOXC1* and *FOXF1* and downregulation of *FOXO3*, *FOXOA1*, and free-radical scavenging enzyme *CAT* (catalase) in myelomatous bones. Initially it was suggested that oxidative stress antagonizes Wnt signaling in osteoblast precursors by diverting β-catenin from T cell factor-mediated transcription to FOXO-mediated transcription [52]. FOXO transcription factors defend against oxidative stress by activating genes involved in free radical scavenging and apoptosis [53], which seems to be indispensable for bone homeostasis. Furthermore, decreasing oxidative stress levels normalizes bone formation and bone mass in mice lacking FoxO1 specifically in osteoblasts [54]. These studies suggest that PTH reduced levels of oxidative stress in myelomatous bones, directly or indirectly, by reducing myeloma tumor burden and that regulation of bone remodeling by FOXO transcription factors depends on the physiological setting. Our data also suggest that other forkhead-related transcription factors, such as *FOXC1* and *FOXF1*, may be involved in osteoblast apoptosis and bone homeostasis.

Although PTH has been shown to upregulate expression of ephrinB2 (*EFNB2*) and to induce activation of ephrinB2/EphB4 forward signaling in murine osteoblasts [55], our GEP and qRT-PCR results showed insignificant increased expression of *EFNB2* following PTH treatment. We previously demonstrated that *EFNB2* and *EPHB4* are downregulated in osteoblast progenitors from patients with MM [56], partially explaining the lack of significant upregulation of *EFNB2* in the current study.

PTH treatment affected expression of genes regulating bone remodeling through signaling pathways other than Wnt signaling. Upregulation of angiopoietin 1 and 2 and angiopoietin-like 2 is consistent with recent reports showing that angiopoietin 1 receptor, Tie2, is upregulated in differentiating osteoblasts and that angiopoietin 1 promotes bone formation [57]. Upregulation of *FGFR1* and *FGFR2* is consistent with previous studies and supports findings that FGF-2 signaling is critically important in the bone-anabolic effects of PTH [58]. Upregulation of *TGFB2*, *TGFBR1*, *SMAD6*, and target genes of TGF (*ID3* and *ID4*) strongly suggests a role for this signaling pathway in mediating the effects of PTH on bone formation and on coupling bone resorption to bone formation [59], [60]. Upregulation of *PDGFA* and *PDGFRA* by PTH treatment suggests involvement of this signaling pathway in the bone-anabolic effects of PTH and supports the notion of using PDGF as a therapeutic agent in treating bone loss associated with aging and fracture healing [61]. Taken together, these results strongly suggest that PTH exerts bone anabolism in myelomatous bone through activation of multiple signaling pathways.

GEP analyses also revealed altered expression of a group of G protein-coupled receptors (e.g., *GPR158*) and phosphatase-related genes (e.g., *PTPRD*), but the functional association between PTH and these factors has yet not been elucidated.

### Effects of PTH on Bone Marrow Microenvironment in MM

Despite the upregulation of RANKL by PTH, we did not observe differences in the numbers of osteoclasts or in expression levels of specific osteoclast markers (e.g., calcitonin receptor and cathepsin K). This phenomenon is not surprising. Lindsay et al [62] described a significant increase in bone formation after one month of PTH treatment but no difference in the eroded perimeter or the osteoclast perimeter compared with controls. The apparent discrepancy could be the result of new bone that is both spatially and temporally unrelated to prior resorption or of bone formation extending to quiescent surfaces adjacent to the original resorption cavity [63]. In experimental and clinical osteoporosis, increased bone formation without increased bone resorption often occurs in the initial stages of response to PTH, whereas catabolism occurs within the context of increased remodeling after approximately 6 months [23]. Thus, the effects of long-term (>6 months) PTH treatment on MM bone disease should be carefully examined. Alternatively, PTH may be given in short cycles or in combination with antiresorptive agents to maximize the bone-forming effects and minimize potential proresorptive effects [23].

The GEP data provided important insights into molecular mechanisms that mediate reduced myeloma growth after PTH treatment. This hormone elicited marked increases in bone formation and increased numbers of mature osteoblasts, which express high levels of potential anti-tumor factors that include decorin, lumican, and CYR61. We recently demonstrated that mature osteoblasts negatively affect growth of myeloma cells [15] and that this effect is partially mediated through production of decorin [64]. Treatment with PTH had no effect on expression of myeloma cell growth factors, such as IL-6 and IGF-1, and PTH treatment did not stimulate myeloma growth in any of the experiments, even though we observed upregulation of *VEGFB* and *HGF*. In addition to suggesting that PTH treatment may lessen oxidative stress in myelomatous bone, the GEP data indicate upregulation of anti-inflammatory factors, such as *TNFAIP6* and *CXCL14*, and downregulation of inflammatory factor *AIF1*. Recent study suggests that *TNFAIP6* (also known as TSG-6) is an important anti-inflammatory factor that mediates improvement of myocardial infarction by systemic mesenchymal cell cytotherapy [65]. Whereas *CXCL14* suppresses tumor growth [66], *AIF1* seems to promote tumor cell proliferation [67]. These findings suggest that reduced inflammatory conditions contribute to PTH control of myeloma cell growth.

### Therapeutic Implications

Current standard management for MM bone disease is limited to reducing tumor burden and treatment with bisphosphonates, and also often includes treatment with dexamethasone, a steroidal component that induces osteoporosis by reducing the life span of osteoblasts [68]. Intriguingly, treatment with PTH has been shown to counteract the adverse effects of glucocorticoids on bone formation and strength [69]. Bortezomib, the first proteasome inhibitor clinically approved for treating MM, stimulates bone formation in our experimental model [70] and in patients with MM [71]. Our current study suggests that combining treatments with PTH may abrogate dexamethasone-induced osteoporosis and act synergistically with proteasome inhibitors to stimulate bone formation and repair bone lesions in MM.

Collectively, the data presented here showed that PTH promotes bone formation in myelomatous bone by activating multiple distinct molecular pathways, of which Wnt signaling seems to play a major role. *In vitro*, PTH has no direct on effect on growth of myeloma cells, but *in vivo* PTH treatment indirectly attenuated MM progression by stimulating osteoblastogenesis and increasing osteoblast production of anti-myeloma factors, and by minimizing oxidative stress and inflammatory conditions in myelomatous bone. Our study supports the notion that MM and its associated bone disease are negatively impacted by alterations in the bone marrow microenvironment induced by osteoblast-activating agents.

## Materials and Methods

### Primary Myeloma Cells

The Institutional Review Board Committee of the University of Arkansas for Medical Sciences (UAMS) has specifically approved this study. Myeloma cells were obtained from heparinized bone marrow aspirates from patients with active MM during scheduled clinic visits. Signed UAMS Institutional Review Board–approved informed consent forms are kept on record. Pertinent patient information is provided in Table 1. Myeloma plasma cells were purified using CD138 immunomagnetic bead separation as previously described [39].

### Myeloma Cell Lines

The BN and Hg myeloma cell lines were established in our laboratory, as previously described [38]. Briefly, primary myeloma cells engrafted in SCID-rab or SCID-hu mice were sequentially passaged into newly constructed animal hosts. BN cells also were grown slowly in coculture with stromal cells and were infected with lentiviral particles containing a luciferase/EGFP construct [38]. The Hg myeloma cell line does not grow *in vitro*, either alone or in coculture with stromal cells, and is maintained by passaging in SCID-rab or SCID-hu mice. The myeloma cell lines CAG and ARP1 were previously established in our institute and grow independently *in vitro* in RPMI 1640 medium supplemented with 10% fetal bovine serum (FBS) and antibiotics.

### Animal Models and Drug Treatment

SCID-hu and SCID-rab mice were constructed as previously described [34], [35]. Animals were housed and monitored in the Department of Laboratory Animal Medicine facility at the University of Arkansas for Medical Sciences. The Institutional Animal Care and Use Committee approved all experimental procedures and protocols (Assurance Number A3063-01, File 2779). Myeloma growth was determined by measuring hIg in mice sera as previously described [35], [36]. Live-animal imaging was performed as previously described [38].

Myelomatous SCID-rab mice were subcutaneously injected with saline or with PTH 1-34 (Bachem California, Torrance, CA) (80 µg/kg/day in 0.9% saline, 0.01 mM 2-ME, 0.1 mM acetic acid) [20], [43] for the indicated period of time. To determine whether increased bone formation affected MM development, SCID-rab mice were pretreated with PTH or saline (as described above) throughout the experimental period. Four weeks after pretreatment with saline or PTH, hosts were injected with the BN myeloma cell line [38], [70] (six mice/group) or with primary myeloma cells from one of three patients (three mice/group for each patient's cells). Tumor growth was monitored for 8–12 weeks or until control mice reached high tumor burden.

### Immunohistochemistry and Histochemistry

Rabbit bones were fixed in 10% phosphate-buffered formalin for 24 hours. Rabbit bones were further decalcified with 10% (wt/vol) EDTA, pH 7.0, and embedded in paraffin for sectioning. Sections (5-µm) were deparafinized in xylene, rehydrated with ethanol, and rinsed in saline and then underwent antigen retrieval by microwave. Sections were immunohistochemically stained for osteocalcin or histochemically stained for TRAP as previously described [15], [34].

### Histomorphometric Analyses

For static histomorphometry, decalcified implanted bone sections were stained with hematoxylin and eosin in most experiments; in some experiments, undecalcified bone sections were stained with Masson's trichrome and used for analysis. Images of the trabecular area were obtained with a 10× objective using an Olympus BH2 microscope (Olympus, Melville, NY). Images were acquired using a SPOT2 digital camera (Diagnostic Instruments, Sterling Heights, MI) and were processed; a total of five images were obtained per section. BV/TV, Tb.Th, and Tn.N were measured using Osteometrics software (Osteometric, Atlanta, GA).

For dynamic histomorphometry, indicated mice were treated with saline or PTH for 3 weeks and then were intraperitoneally injected with tetracycline (30 µg/kg, Sigma-Aldrich) 10 days or 3 days before sacrificing. Undecalcifed implanted bones were processed as previously described [72], and images demonstrating typical single- and double-labeled areas were obtained with a 20× objective.

### Effects of PTH on Cell Growth *In Vitro*

Saos-2 osteosarcoma cells were maintained in McCoy's medium supplemented with 15% FBS and used as a positive control for response to PTH [41]. Cells from the MDA-231 breast cancer line variant, which reportedly do not express PTH1R [73], were maintained in α-MEM medium supplemented with 10% FBS and insulin (10 µg/ml) and were used as a negative control for PTH1R expression (see below). ARP1 and CAG myeloma cell lines were established in our institution and were maintained in RPMI 1640 medium supplemented with 10% FBS. Primary myeloma plasma cells were selected from bone marrow of patients with MM using CD138 immunomagnetic bead separation [12]. The examined cell lines were cultured in 96-well plates (20–25×10^3^ cells/well in 100 µl) for 72 hours, and primary myeloma plasma cells were cultured in 24-well plates (0.5×10^6^ cells/well in 1 ml) for 24 hours in the presence and absence of PTH (1–100 nM). In a subset of experiments, CAG and Saos-2 cells were cultured similarly in serum-free conditions for 72 hours. The effects of PTH on cell growth were determined using MTT (3-(4,5-dimethylthiazol-2-yl)-2,5-diphenyltetrazolium bromide) assays. Expression of PTH1R and PTH2R in indicated cells was determined by qRT-PCR (see below).

### Global GEP

Myelomatous implanted human bones were removed from SCID-hu mice 2 hours after the last injection of saline or PTH and were used for GEP analyses; a total of five profiles were analyzed in each group. After removal from the hosts, bones were immediately snap-frozen in liquid nitrogen. Frozen bones were ground thoroughly in liquid nitrogen by using a mortar and pestle. The tissue powder and liquid nitrogen were decanted into a cooled tube, and the liquid nitrogen was allowed to evaporate. RNA extraction was then performed using RNeasy Fibrous Tissue Mini Kit according to the manufacturer's instructions (Qiagen Inc., Valencia, CA).

As previously described, GEP was performed using the Affymetrix U133-Plus microarray, which contains approximately 54,000 genes (Affymetrix, Santa Clara, CA) [39]. Expression levels of individual probe sets in the PTH treatment group were compared with those in the control group. A difference in expression of a probe set was identified as significant if (a) the comparison between the two groups had p<0.05 using Student's *t*-test; (b) mean signal was >250 in the PTH group when assessing upregulated genes and >250 in the control group when assessing downregulated genes; and (c) the comparison had an absolute fold change >2. All the GEP data is MIAME compliant and the GEP raw data has been deposited in the MIAME-compliant database, GEO (Gene Expression Umnibus).

### qRT-PCR

Total RNA (1 µg) from each sample was reverse-transcribed with the SuperScript III First-Strand Synthesis SuperMix for qRT-PCR (Invitrogen Corp., Carlsbad, CA). The qRT-PCR was performed with the TaqMan gene expression assay on an ABI Prism 7000 sequence analyzer according to the manufacturer's recommended protocol (Applied Biosystems, Foster City, CA). Reverse-transcribed RNA (10 ng) was amplified by using the TaqMan Universal PCR Master Mix and TaqMan gene expression assays (ID HS99999905_m1 for GAPDH as an endogenous control, ID Hs00970627_m1 for EFNB2, ID Hs 00174752_m1 for EPHB4, ID Hs01029144_m1 for ALPL, ID Hs01002399_m1 for BMP7, ID Hs00154192_m1 for BMP2, ID Hs00156229_m1 for CALCR, ID Hs00370383 for DCN, ID Hs00158940_m1 for LUM, ID Hs00370078 for BMP4, ID Hs00164004_m1 for COLL1A1, ID Hs00166156_m1 for CTSK, ID Hs00183740_m1 for DKK1, ID Hs01587813_g1 for BGLAP, ID Hs00231692_m1 for RUNX2, ID Hs00228830_m1 for SOST, ID Hs00985639_m1 for IL6, ID Hs01547656_m1 for IGF1, ID Hs00900358_m1 for TNFRSF11b, ID Hs00243519_m1 for TNFSF11).

Each reaction was run in duplicate. The comparative threshold cycle (CT) method was used to calculate the amplification fold, as specified by the manufacturer.

### Statistical Analyses

All values are expressed as mean±SEM. The effect of treatment on BMD, myeloma tumor burden, osteoblast and osteoclast numbers, and GEP were analyzed using Student's *t*-test in experiments with Hg and BN myeloma cell lines and using Student's paired *t*-test in experiments with primary myeloma cells.

## Supporting Information

Table S1 **Genes whose expression was significantly upregulated or downregulated by PTH treatment in whole human bone from SCID-hu mice.** SCID-hu mice engrafted with Hg myeloma cells were treated with saline or PTH for 4 weeks. Mice were sacrificed 2 hours after the last injection. RNA extracted from the whole myelomatous human bone (five bones/group) was subjected to global gene expression profiling. Positive (POS) or negative (NEG) expression of those genes in Hg myeloma cells is indicated to assess cellular source of these genes (e.g., microenvironmental and/or tumor cells).
(XLS)
